# The Association Between Psychological Symptoms and Self-Reported Temporomandibular Disorders Pain Symptoms in Children and Adolescents

**DOI:** 10.3389/froh.2021.675709

**Published:** 2021-11-19

**Authors:** Amal Al-Khotani, Dalia E. Meisha, Samaa Al Sayegh, Britt Hedenberg-Magnusson, Malin Ernberg, Nikolaos Christidis

**Affiliations:** ^1^Dental Department, East Jeddah Hospital, Ministry of Health, Jeddah, Saudi Arabia; ^2^Scandinavian Center for Orofacial Neurosciences (SCON), Huddinge, Sweden; ^3^Division of Oral Diagnostics and Rehabilitation, Department of Dental Medicine, Karolinska Institutet, Huddinge, Sweden; ^4^Department of Dental Public Health, Faculty of Dentistry, King Abdulaziz University, Jeddah, Saudi Arabia; ^5^Department of Orofacial Pain and Jaw Function, Eastman Institutet, Folktandvården Stockholm AB, Stockholm, Sweden

**Keywords:** pain, anxiety, depression, somatic, TMD (temporomandibular disorders)

## Abstract

**Background:** Several studies have reported an association between temporomandibular disorder pain (TMD-P) and emotional disorders in children and adolescents. However, no studies have reported if self-reported TMD-P in Saudi Arabia is associated with psychosocial symptoms. Therefore, the current study aimed to evaluate the association between self-reported TMD-P with depression, anxiety and somatic problems in children and adolescents in Saudi Arabia. The hypothesis was that there is an association between self-reported TMD-P and psychological symptoms among children and adolescents.

**Materials and Methods:** The included participants were randomly selected boys and girls aged between 10 and 18 years, with a mean (SD) age of 14.0 (2.3) years. Out of 633 children and adolescents that were invited to participate, 509 voluntarily agreed to participate, and 466 completed all questionnaires. The questionnaires included items retrieved from the Youth Self Report (YSR) and Axis II of the Research Diagnostic Criteria for TMD (RDC/TMD) besides demographic data, medical history, and presence of oral parafunctions. To assess the presence of self-reported TMD-Pain, each participant was verbally asked two validated questions regarding the presence of TMD-P and dysfunction (2Q-TMD).

**Results:** Self-reported TMD-P in children and adolescents was significantly associated with anxiety, depression, somatic symptoms, and social problems (*P* < 0.0001). Further, the frequencies of anxiety, depression, and somatic disorders were more evident among children and adolescents who suffered from TMD-P (*P* < 0.0001). The odds of reporting TMD-P in children and adolescents was 1.4 times for border line and clinical diagnosis scores for anxiety and withdrawal depression domains, and 2.6 times for the somatic symptoms' domains. However, in the multiple regression model after controlling for possible confounders, only somatic symptoms and social scores were significant. Moreover, self-reported TMD-P was twice as prevalent among girls compared to boys.

**Conclusion:** This study reports a significant association between psychosocial burden and presence of self-reported TMD-Pain, with a stronger impact on girls than boys. There were significantly higher number of participants with self-reported TMD-P reporting a poor oral and general health. In addition, self-reported TMD-P was higher among those with borderline and clinically diagnosed anxiety/depression scores. Based on this finding, the current study supports that an early approach and recognition of children and adolescents with anxiety, depression, somatic symptoms, and TMD problems. This could result in a lesser burden for these children and adolescents both in regard to pain and psychosocial implications with increased quality of life.

## Introduction

It is well-documented that pain disorders, such as the pain in orofacial or temporomandibular joint (TMJ) regions, are comorbid with biopsychosocial factors [1–3]. Temporomandibular disorders (TMD) are a heterogeneous group of symptoms including signs and symptoms with different degrees which affect the musculoskeletal system in the face and TMJ area [4]. Studies have shown that the prevalence of TMD signs and symptoms range between 20 and 34% [5–8]. It is worth mentioning that TMD patients respond more to stressors in the masticatory muscles than in other muscle systems [9]. One cannot ignore the importance of this area since it includes a complex mechanism while the person is chewing and talking. Therefore, pain or dysfunction in this part will negatively affect the children's and adolescents' well-being. When left untreated, patients who reported TMD pain (TMD-P) in adolescents have a threefold risk of TMD-P in adulthood [10].

TMD-P can be comorbid with somatic painful complaints such as headache, neck pain, and back pain [10–12]. Non-painful complaints can also co-occur with TMD-P such as social impairment, anxiety, depression, and low school performance [10, 12–15]. In addition to comorbidities, the challenge clinicians encounter is the diagnosis of TMD due to its special anatomic location and proximity to complex structures in the head and neck area. This unique location can lengthen the journey of reaching a definite TMD diagnosis which in turn increases the patient's stress and anxiety.

Anxiety can be defined as a person's expected emotional condition in certain situations [16]. It is considered one of the internalizing psychosocial disorders that commonly affect children and adolescents suffering from pain [17]. Recently, it has been reported that the prevalence of anxiety and its consequent depression and somatic complains are increasing among children and adolescents [18]. Relying on its prevalence, many studies elaborated the possible causes of anxiety in children and adolescents such as chronic pain, physical illness, puberty, and health problem secondary to a disease or syndrome [18]. Accordingly, patients usually ease their stress by using their stomatognathic system, in turn this leads to increase in the secretion of cortisol level in saliva [19]. Consequently, the secretion of cortisol level leads to muscular tension and developing of parafunctional habit including bruxism, teeth grinding, and clenching [20]. As a response, psychophysiological symptoms, which indicate an individual muscular reaction, might appear secondary to stressful circumstances [21]. Long term, untreated anxiety can progress into depression and somatic disorders such as TMD-P [10, 22, 23].

In Saudi Arabia, it has been shown that more than half of adolescent boys have at least one emotional disorder (depression, anxiety, and stress) [24]. Other studies found more than one quarter of children and adolescents diagnosed with one or more TMD problems [8]. Together, several studies have reported an association between TMD-P and emotional disorders in children and adolescents [11, 15, 23]. However, no study, to the authors knowledge, has reported self-reported TMD-P in Saudi Arabia associated with psychosocial symptoms. Therefore, the current study aimed to evaluate the association between self-reported TMD-P and depression, anxiety and somatic problems in children and adolescents in Saudi Arabia. The hypothesis was that there is an association between self-reported TMD-P and psychological symptoms among children and adolescents.

## Materials and Methods

This cross-sectional study was conducted between January 2014 and March 2014 and was part of a project with several studies carried out in the city of Jeddah, Saudi Arabia [8, 15, 23]. The study was approved by the local ethical committee at the Department of Medical Study and Research, Ministry of Health, Jeddah, Saudi Arabia following the Declaration of Helsinki guidelines. All participants received both written and verbal information and gave both verbal and parental written consent prior to inclusion.

### Participants

The only inclusion criterion for the participating children and adolescents was being at the age between 10 and 18 years. To obtain results that could be generalizable the present study did not have any exclusion criteria; thus, all plausible participants were invited. However, the participants of the current study were randomly selected from different schools in Jeddah city. This randomization process was conducted as follows:

1. The city of Jeddah was divided into the five predefined regions: North, South, East, West, and Central.
2. The inclusion of schools was based on a predefined set of schools, as clustered by the ministry of education, in order to obtain a representative sample of the city of Jeddah.
3. The educational system in Saudi Arabia is based on single-sex schools (i.e., boy-schools vs. girl-schools).
4. Two boy-schools and two girl-schools were randomly selected from each region. This randomization was performed with a computer-based application (www.randomization.com) by one of the researchers (NC) who did not participate in data collection.
5. One class from each school (having on average 30 pupils) was also randomly selected using a simple sampling method. This simple sampling was done by using a bucket with the titles of the school classes from the specific school from which a dental assistant not participating in the data collection drew one class.

### Study Protocol(s)

As this study is part of a larger project, this paper only presents the Axis II of the Research Diagnostic Criteria for TMD (RDC/TMD). The clinical diagnoses, based on Axis I of the RDC/TMD, are presented in another study [8]. Due to the single-sex education system in Saudi Arabia, two protocols were used: one for girls and one for boys. To reduce the impact of using two protocols and different locations/surroundings during the examinations only one examiner (AA-K) did all examinations, and the same equipment was used in both examination facilities. Furthermore, collected data included demographic data (including ethnic and socioeconomic background), medical history, headache, and presence of oral parafunctions, i.e., the biopsychosocial aspects of TMD.

#### Protocol for Girls

One day before their clinical examination, all girls and their parents received information about the purpose of the study and a brief explanation of the questionnaires they were asked to fill in. On the day of clinical examination (Axis I of the RDC/TMD), the participating girls were examined in their schools, during the ordinary school day, using a mobile dental chair in the nurse's room. Also, the girls completed the official Arabic version of the YSR (YSR; licensed from ASEBA/Research Centre for Children, Youth & Families, University of Vermont, Burlington, USA).

#### Protocol for Boys

One appointment was offered to each presumable participant. The boys were accompanied by a parent/guardian to the clinic. To reduce the risk of parent/guardian interference the parents/guardians were asked to wait outside the clinical room. However, if the parent/guardian insisted to attend together with their son, they were asked to remain passive during the entire session. The participating boys were examined at a dental clinic, at the primary health care center of that region.

All boys were examined according to Axis I of the RDC/TMD. Their parents received information about the purpose of the study and a brief explanation of the questionnaires they were asked to fill in. This questionnaire is the Arabic version of the YSR (YSR; licensed from ASEBA/Research Centre for Children, Youth & Families, University of Vermont, Burlington, USA).

### Self-Reported Temporomandibular Disorder Pain

To assess the presence of self-reported TMD-P, each participant was verbally asked two validated questions regarding (1**)** the presence of TMD-P, and (2**)** the presence of jaw dysfunction once a week or more often [25, 26]. In young patients, the reliability values range from acceptable to excellent in correlation to Axis II of the RDC/TMD [27, 28]. The first question was “*Do you have pain in the temple, face, temporomandibular joint, or jaws once a week or more?,”* while the second question was “*Do you have pain when you open your mouth wide or chew once a week or more?.”* If the participant marked “yes” to one or both of the two questions was regarded as TMD-P. Thus, the participants were categorized into two groups: TMD-P and no TMD-P.

### Questionnaires

#### Research Diagnostic Criteria for Temporomandibular Disorders (RDC/TMD)

The RDC/TMD is a dual diagnostic tool (**Axis I and II**) that is reliable and used for children and adolescents [27].

- The data from **Axis I**, which is the diagnostic algorithm for clinical TMD diagnoses, were presented previously [23].
- The **Axis II** included, Symptom Checklist-90-Revised (SCL-90-R), Graded Chronic Pain Scale (GCPS), and Jaw Disability Checklist (JDC).
- The SCL-90-R is not validated for children and adolescents younger than 13 years of age [29], therefore it was replaced with the Arabic version of the YSR.
- The data obtained from GCPS and JDC was presented elsewhere [8].

#### Youth Self Report (YSR)

This Arabic version of YSR is validated and reliable [30] and was licensed from the ASEBA/Research Center for Children, Youth & Families, University of Vermont, Burlington, VT USA [31]. The YSR is a scale that shares the same purpose as SCL-90-R but is constructed for children and adolescents of all school ages [32].

The YSR contains two main domains including 112 problem statements [32]. The first domain is the Problem Checklist, and the second domain is the Social Competence.

##### The Problem Checklist Domain

This domain assesses emotional and behavioral functioning that consists of 109 statements. Those statements explicate five major clusters: (1) anxiety; (2) depression; (3) somatic complaints; (4) aggressive disorders; and (5) social and attention problems. These major clusters are grouped into three subscales: (a) broad-band internalizing and externalizing; (b) eight narrow-band syndromes; and (c) DSM-oriented scales [32].

This study focuses on the first three narrow-band syndromes, which are: (1) Anxious/Depressed; (2) Withdrawn/Depressed; (3) Somatic Complaints. The other five narrow-band syndromes, as well as the DSM-oriented scales, are already presented in a previous study [23]. The YSR Anxious/Depressed syndrome scale indicates the internalizing problems that can lead to suicidality. Whereas, Withdrawn/Depressed syndrome indicates the internalizing problems that can be known as a protective factor against negative thinking including suicidality [33].

Due to cultural considerations, three statements about sexual problems were removed in the current study. This was also done in a previous study regarding TMD-P in an adult cohort in Saudi Arabia [34]. A licensed software scoring program (ASEBA^TM^ version 9.1, Burlington, VT, USA) was used to calculate and present percentiles and T-scores for all subscales and syndromes. The normal T-score range for all syndromes is between 50 and 64, the borderline clinical T-score range is between 65 and 69, while the clinical T-score range is between 70 and 100 [35].

##### The Social Competence Domain

This domain measures the social competence and physical activities that comprises of seven statements covering three areas: (1) social relations; (2) physical activities; and (3) the mean of self-reported academic performance [32]. The same licensed software scoring program (ASEBA^TM^ version 9.1, Burlington, VT, USA) was used for this domain to calculate and present percentiles and T-scores for all activities and social competencies. The normal T-score range for all syndromes is between 36 and 65, the borderline clinical T-score range is between 32 and 35, while the clinical T-score range is between 20 and 31 [35].

### Statistical Analysis

Sample size calculation was done using G-power (Version 3.1.9.3). Based on a previous study [23], results, the prevalence of anxiety was reported to be 24.1 and 18.4% among those diagnosed with TMD vs. no TMD. Assuming an alpha of 0.05 and a power of 80%, the estimated minimum sample size was 396 subjects for this study.

Statistical analyses were performed using the statistical software: IBM SPSS Statistics (Version 24, Armonk, NY: IBM Corp). The dependent variable was the self- reported TMD-P; categorized as a dichotomous variable no/yes. Demographic data were categorical and included gender, nationality, school grade, parental income, and occupation. Psychosocial scores included Anxiety/Depression score, Withdrawal/Depression score, Somatic Complaints score, Activity Score, and Social Score. Scores were not normally distributed (Kolmogorov-Smirnov *p* < 0.0001), so Mann-Whitney U tests were used to determine if there were any significant differences in psychosocial scores based on self- reported TMD pain status. Logistic regression models were performed to predict which variables were determinants of self-reported TMD-P status at 0.05 level of significance.

## Results

### Study Participants

**Figure 1:**
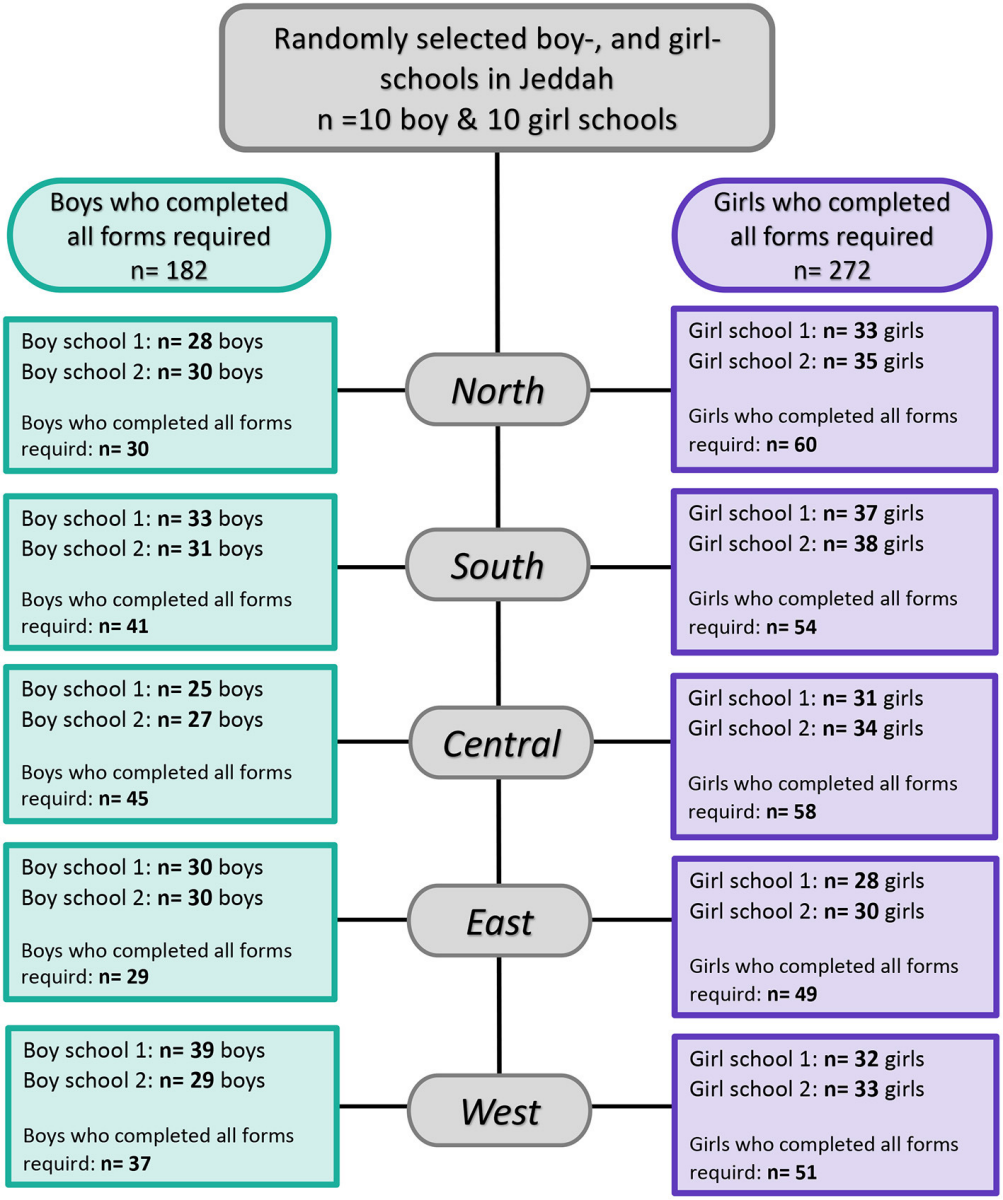
Flowchart illustrating child and adolescent participation in this study among the general population in Jeddah, Saudi Arabia.

Five-hundred nine participants agreed to participate that is equivalent to a response rate of 80.4%. Out of those 509 participants, 466 completed all study questionnaires (Figure 1). Their age ranged from 10 through 18 years with a mean (SD) of 14.0 (2.3) years. They were categorized according to self-reported TMD-P into two groups: no TMD-P and TMD-P. The demographic characteristics for the participants are shown in Table 1. About 60% of participants were female with more than half reporting having less than average income (SR15,000). Among the fathers, 37.6% of the sample were white collar, while 84% of mothers reported being unemployed. There was a significantly higher proportion of participants with self-reported TMD pain reporting poor oral health, general health, and presence of a TMD diagnosis.

**Table 1:** Demographic characteristics grouped according to self-reported temporomandibular pain (TMD-P) among adolescents living in Jeddah, Saudi Arabia (*N* = 466).

	**Total**	**No TMD-P**	**TMD-P**	***P*-value**
	***N* (%)**	***N* (%)**	***N* (%)**	
**Age**				0.6
10–13 years	235 (51.5)	135 (50.6)	100 (52.6)	
14–18 years	221 (48.5)	132 (49.4)	89 (47.1)	
**Gender**				0.05^*^
Boys	182 (40.4)	118 (44.2)	66 (34.9)	
Girls	272 (59.6)	149 (55.8)	123 (65.1)	
**Nationality**				
Saudi Arabian	291 (63.8)	175 (65.5)	116 (61.4)	0.4
Non-Saudi	165 (36.2)	92 (34.5)	73 (38.6)	
**School grade**				0.5
Primary school (Grade 1–6)	213 (41.4)	122 (45.7)	91 (48.1)	
Middle school (Grade 7–9)	139 (27)	87 (32.6)	52 (27.5)	
High school (Grade 10–12)	104 (20.2)	58 (21.7)	46 (24.3)	
**Parental income**				0.8
Below average	233 (52.6)	133 (51.8)	100 (53.8)	
Average	149 (33.6)	90 (35)	59 (31.7)	
Above average	61 (13.8)	34 (13.2)	27 (14.5)	
**Living with parents**				0.3
No	28 (7.2)	19 (8.4)	9 (5.5)	
Yes	362 (92.8)	207 (91.6)	155 (94.5)	
**Father's occupation**				0.2
White collar	163 (37.6)	98 (38.6)	65 (36.3)	
Blue collar	149 (34.4)	86 (33.9)	63 (35.2)	
Gold collar	44 (10.2)	28 (11)	16 (36.4)	
Gray collar	54 (12.5)	25 (9.8)	29 (16.2)	
No collar	23 (5.3)	17 (6.7)	6 (3.4)	
**Mother's occupation**				
White collar^*^	46 (11.3)	32 (13%)	14 (8.8)	0.4
Blue collar^*^	5 (1.2)	3 (1.2	2 (1.3)	
Gold collar^*^	5 (1.2)	3 (1.2)	2 (1.3)	
Gray collar^*^	8 (2)	7 (2.8)	1 (0.6)	
No collar^*^	343 (84.3)	202 (81.8)	141 (88.1)	
**General health**				
Excellent	273 (60.3)	180 (67.7)	93 (49.7)	<0.0001^*^
Good	149 (32.9)	75 (28.2)	74 (39.6)	
Poor	31 (6.8)	11 (4.1)	20 (10.7)	
**Oral health**				0.001^*^
Excellent	130 (28.8)	93 (35.1)	37 (19.8)	
Good	243 (53.8)	136 (51.3)	107 (57.2)	
Poor	79 (17.5)	36 (13.6)	43 (23)	
**TMD-P**				
No	332 (72.8)	240 (89.9)	92 (48.7)	<0.0001^*^
Yes	124 (27.2)	27 (10.1)	97 (51.3)	

*Data are presented as n (%)*.

* *Statistical significance (Chi-square tests; P ≤ 0.05)*.

*White collar: jobs including office setting (as secretary, marketing, company employer, academia)*.

*Blue collar: jobs including manual labor (as military, manufacturing, sales, fishing, power operation)*.

*Gold collar: Jobs that need high education and skills (as surgeons, engineers, airline pilot, and lawyers)*.

*Gray collar: retired*.

*No collar: unemployed, volunteer*.

### Self-Reported TMD-P and Emotional/Somatic Functioning Scores

**Figure 2:**
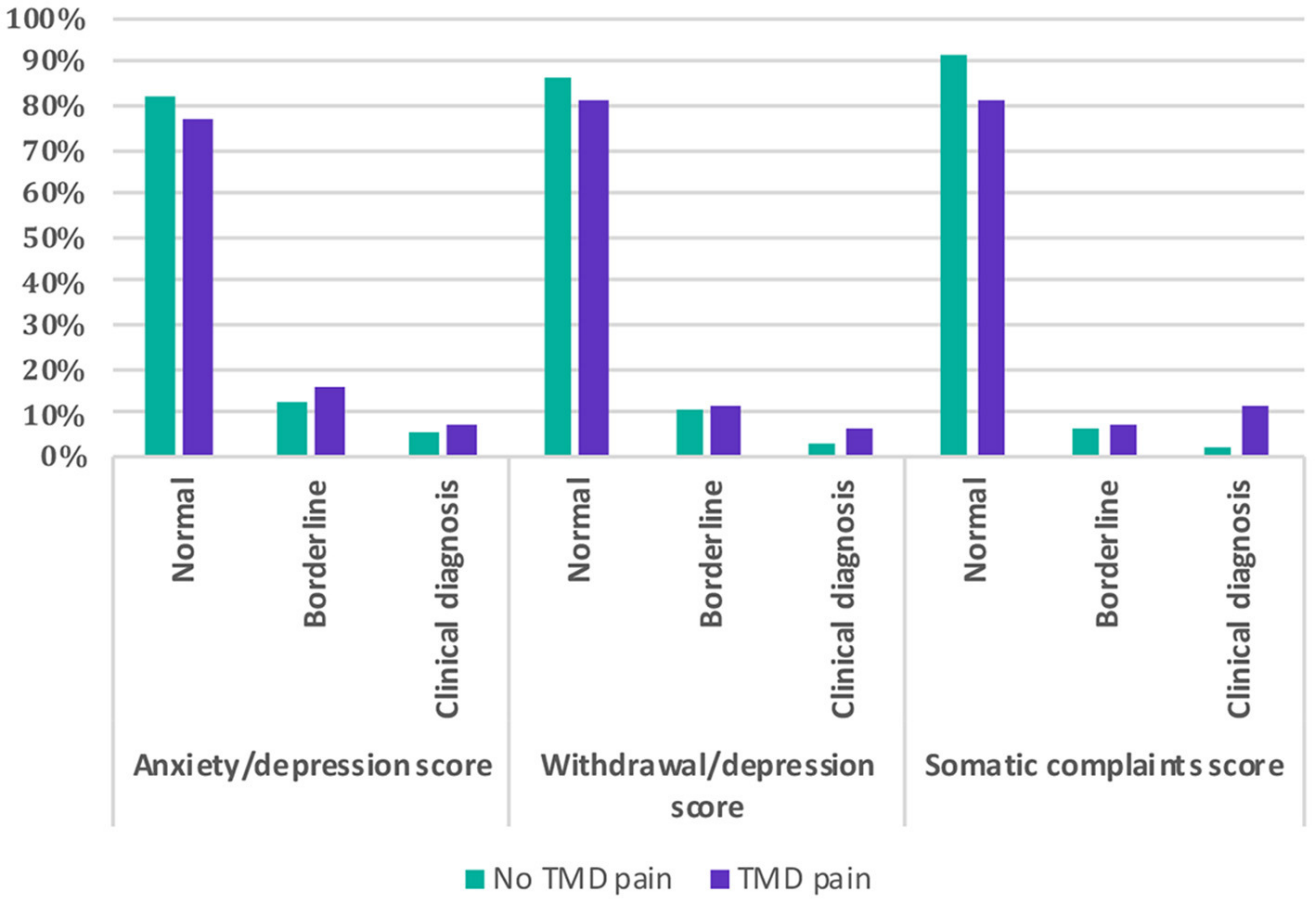
Comparison of psychosocial scores (anxiety/depression, withdrawal/depression, and somatic complaints) among children and adolescents with and without self-reported painful temporomandibular disorders (TMD-P).

Participants reporting TMD-P had significantly higher Anxiety/Depression score, Withdrawal/Depression score, and Somatic Scores compared to those with no reported TMD-P (Table 2). A higher proportion of the participants with self-reported TMD-P were among the borderline and clinically diagnosed categories in terms of Anxiety/Depression score, Withdrawal/Depression score, and Somatic Scores (Figure 2). However, among the three psychosocial score domains, the sum of the frequencies of the borderline and clinical diagnosis scores in the Anxiety/Depression domain was highest among the TMD-P group compared to the other domains. The odds of reporting TMD-P in children and adolescents was 1.4 times for border line and clinical diagnosis scores for anxiety and withdrawal depression domains, and 2.6 times for the somatic symptoms' domains compared to children and adolescents with no TMD-P.

**Table 2:** Self-reported psychosocial scores according to the Youth Self Report (YSR) among children and adolescents living in Jeddah, Saudi Arabia grouped according to the presence of self-reported temporomandibular disorder pain (TMD-P).

**Youth self report**	**No TMD-P**	**TMD-P**	***P*-value**
**Anxiety/depression**			
Mean ± SD	57.5 ± 6.8	59.1 ± 7.7	0.03^*^
Median (IQR)	56 (11)	59 (12)	
**Withdrawal depression score**			
Mean ± SD	56.5 ± 6.3	58.2 ± 7.8	0.02^*^
Median (IQR)	55 (10)	58 (13)	
**Somatic score**			
Mean ± SD	53.8 ± 5.4	58.1 ± 9.5	<0.0001^*^
Median (IQR)	51 (5)	54 (12)	
**Activity score**			
Mean ± SD	32.8 ± 7.6	33.4 ± 8.1	0.8
Median (IQR)	32 (11)	32 (13)	
**Social score**			
Mean ± SD	40.3 ± 41.2	41.2 ± 6.6	0.3
Median (IQR)	41 (10)	41 (10)	

* *Statistical significance (Mann-Whitney U tests; P ≤ 0.05)*.

**Figure 3:**
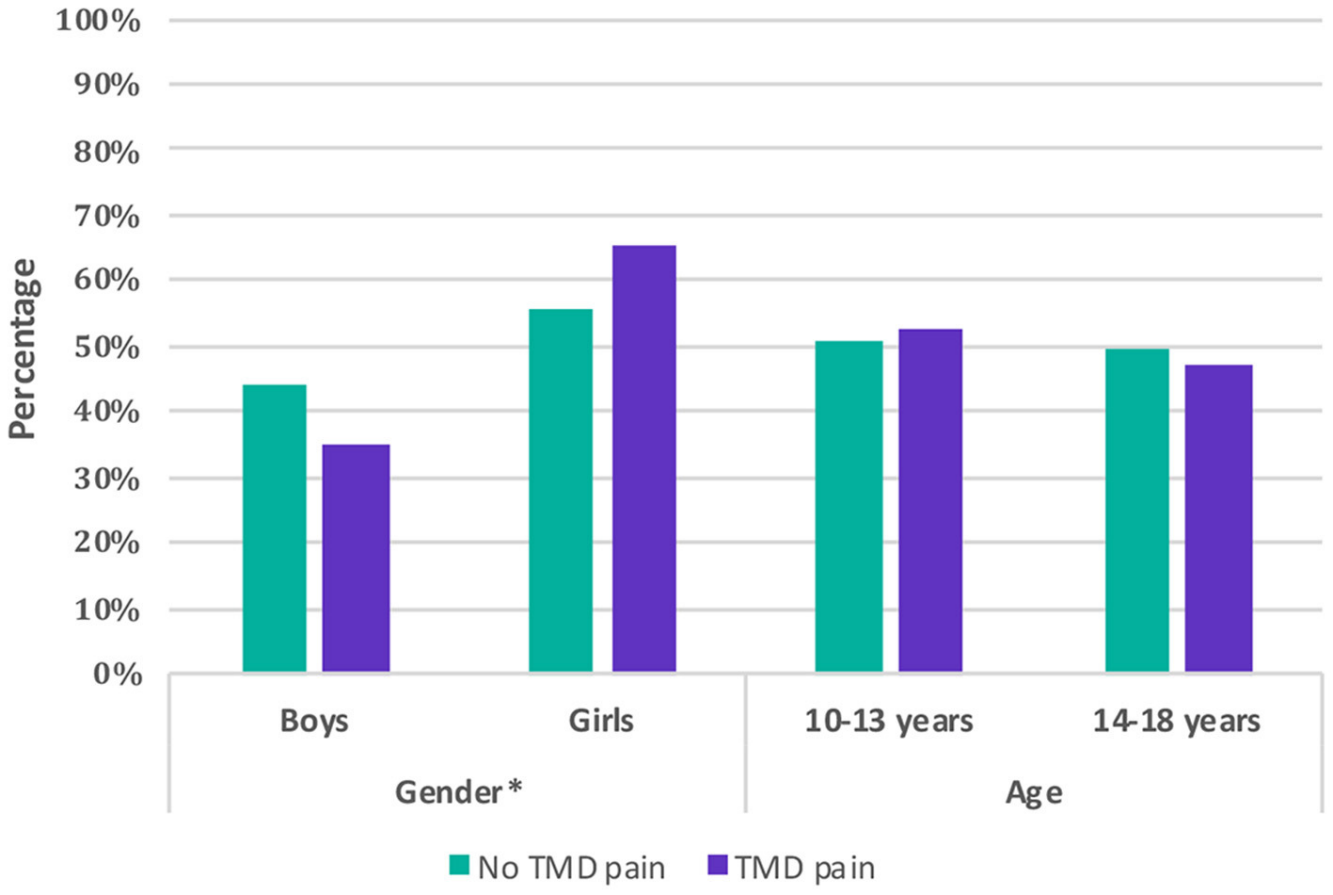
Comparison of children and adolescents with and without self-reported painful temporomandibular disorders (TMD-P) by gender and age.

The subgroup analysis stratified by age showed that adolescents of the age group 14–18 years had a significantly higher Anxiety/Depression score (by 2.94 scores) and Withdrawal/Depression score (by 1.95 scores) compared to the age group 10–13 years. Girls and boys were only different in their Social scores, as girls had lower Social score by 3.88 (Figure 3).

The multivariate logistic regression model showed that having a confirmed TMD diagnosis (OR 8.8, 95% CI 5.0–15.8), being a female (OR 2.4, 95% CI 1.3–4.2), and higher somatic complaints (OR 1.1, 95% CI 1.001–1.1) and social problems scores (OR 1.1, 95% CI 1.01–1.1) were significantly associated with the self- reported TMD-P (Model *P* < 0.0001) (Table 3).

**Table 3:** Logistic regression predicting self- reported TMD pain among adolescents in Jeddah, Saudi Arabia.

	**Adjusted odds ratio**	**OR 95% CI**	***P*-value**
**Gender**			
Male (Reference)			
Female	2.4	(1.3,4.2)	0.003^*^
**Tempromandibular disorders**			
No (Reference)			
Yes	8.8	(5.0,15.8)	0.0001^*^
**Somatic complaint domain**	1.1	(1.001,1.1)	0.0001^*^
**Social score**	1.1	(1.01,1.1)	0.04^*^

*CI, Confidence Interval*.

* *Indicates statistical significance*.

*Hosmer-Lemeshow test p-value is 0.6, R-square 35.2%, Model P < 0.0001*.

## Discussion

Since pain is a subjective feeling affected by the bio-psychosocial model, our study aimed to evaluate the associations between self-reported TMD-P and depression, anxiety, and somatic problems in children and adolescents. The results showed that children and adolescents with self-reported TMD-P reported higher scores for anxiety, depression, and somatic symptoms compared to children and adolescents with no TMD-P complaint. Similar to our results, previous studies have reported that internalizing emotional problems such as depression and anxiety are associated with TMD signs and symptoms and a TMD diagnosis [2, 23, 36, 37]. Furthermore, some studies measured the level of urinary catecholamines in young children and adolescents; they found that those children were not only emotionally stressed but also were at risk of developing TMJ tenderness [38].

Concerning anxiety, depression, and somatic diagnoses, our study showed that a higher proportion of children and adolescents with TMD-P reported Anxiety/Depression than other domains. A recent longitudinal study suggested that TMD-P in children and adolescents was increased three times in adulthood [10]. Taken together, anxiety in adolescents primarily increased their attention to pain. This attention could enhance their pain sensation, which can induce depression or suicidality in the future [39, 40]. In comparison to this explanation, a recent systematic review study found that the suicidal risk is increased two times in adolescents with previous pain experience [41].

The present study showed that self-reported TMD-P was nine times more frequent among those with a confirmed RDC/TMD pain diagnosis. The current results corroborate previous results of a significant association between self-reported TMD-P and the presence of confirmed TMD diagnosis [8, 10]. However, one has to consider that not all children and adolescents with a self-reported TMD-P were diagnosed with a TMD diagnosis using the RDC/TMD Axis one protocol. One explanation could be that children and adolescents reporting TMD-P were suffering from odontogenic pain. Another reason could be that orofacial pain could be a comorbidity to psychological distress or pain from other body parts [1, 21]. These comorbidities might be painful or non-painful [10]. This could be based on the fact that stress and anxiety induce tension-reducing activities such as parafunctional habits. In turn, these tension-reducing habits will affect both teeth and associated muscular structure [21, 42].

Concerning somatic complaint, our study showed that TMD-P is significantly associated with somatic disorders. Similarly, other studies reported headache, backache, and stomachache in children and adolescents with TMD problems [23]. When oral and general health of children and adolescents were controlled for, there was a significant difference between the TMD-P group and the no TMD-P group. However, about one-quarter of the children and adolescents in the TMD-P group reported poor oral health compared to one out of ten children and adolescents from the no TMD-P. An explanation for this significant difference might be that children and adolescents with TMD-P can experience pain-associated challenges such as stressors associated with the period of hormonal changes, their desire to become independent, missing school due to clinical appointments or hospitalizations [43, 44]. Consequently, the level of anxiety and depression could be elevated due to increased stress and worry about their bad teeth and orofacial pain and how this will develop in the future [18]. In turn, depression would negatively affect children's and adolescents' peer relationships. Indeed, our study showed a significant association between social problems and the presence of TMD-P.

Moreover, the present study found a significant association between self-reported TMD-P and female sex. Self-reported TMD-P was twice as frequent among girls than boys. Similarly, Nilsson and co-workers 2001 found a higher prevalence of TMD-P among adolescents girls than adolescents boys [28]. Other studies reported that depression and somatic pain was more significant among adolescent girls than boys [1, 36]. Nevertheless, this association cannot be ignored and can be interpreted in a variety of ways. Such a way could be through stress intensity, which occurs more often among girls than boys [1, 11]. Another study reported that pain among girls is aggravated by medical disorders and psychosocial as well as emotional factors [45]. Another interpretation can be female sex-hormone and its relation to TMD-P [46]. However, many studies showed that female sex-hormones are considered a predictive factor for development TMD [46, 47]. Hence, alterations (polymorphisms) in the catechol-O-methyl-transferase (COMT) genotype, was shown to be responsible for TMD-pain onset, and therefore can be one explanation since it has been proven that the risk of emerging TMD increases when COMT activity decreases [48]. In contrast to girls, boys tend to distract themselves from pain by physical activities, and in turn, they recover more rapidly from TMD symptoms than girls, which might be an explanation [47].

This study has numerous strengths. One strength is data collection through the rigor randomization process. Hence, this process would permit the generalizability of the results to the residents of Saudi Arabia. The use of two validated questions (2Q-TMD) is another strength of our study. Also the use of the self-reported YSR in the current research is another strength since it is a valid and reliable instrument [35]. Moreover, the one examiner was calibrated to a reference standard examiner performed all clinical examinations using the validated RDC/TMD examination, which allows for comparison with other studies using the same system. Another strength is that only one examiner AA-K did the clinical examinations according to Axis I of the RC/TMD and was also the one who asked and explained the two TMD-questions.

One could argue that the unequal numbers between boys and girls, with a higher number of dropouts among the boys, can be seen as a limitation of this study. This was probably due to the study set-up with different settings for boys and girls. The girls were examined in the school nurse's room using a mobile dental chair which made the examination very accessible. The boys, on the other hand, had to go to a dental clinic at their primary health care center, which could explain the higher number of dropouts. This difference could not be avoided due to the nature of the school system in Saudi Arabia.

We concluded that there is a significant association between psychosocial problem and presence of TMD-P in children and adolescents in Saudi Arabia. There was a significantly higher number of children with poor oral and general health the reported TMD-P. Based on this finding, an early approach and recognition of children and adolescents with anxiety, depression, somatic symptoms and TMD problems could result in a lesser burden for them and in turn increase quality of life.

## Data Availability Statement

The raw data supporting the conclusions of this article will be made available by the authors, without undue reservation.

## Ethics Statement

The studies involving human participants were reviewed and approved by the Local Ethical Committee at the Department of Medical Study and Research, Ministry of Health, Jeddah, Saudi Arabia. Written informed consent to participate in this study was provided by the participants' legal guardian/next of kin.

## Author Contributions

AA-K, BH-M, ME, and NC: conceptualization. AA-K, ME, and NC: methodology and validation. AA-K, DM, SA, NC, and ME: software. AA-K, DM, SA, and NC: formal analysis. AA-K: investigation and funding acquisition. AA-K, DM, SA, BH-M, ME, and NC: resources and writing (review and editing). AA-K, DM, and SA: data curation. AA-K and DM: writing (original draft preparation). AA-K and NC: visualization and project administration. BH-M, ME, and NC: supervision. All authors contributed to the article and approved the submitted version.

## Funding

The current study was financially supported by a grant from Ministry of Health, Saudi Arabia.

## Conflict of Interest

The authors declare that the research was conducted in the absence of any commercial or financial relationships that could be construed as a potential conflict of interest.

## Publisher's Note

All claims expressed in this article are solely those of the authors and do not necessarily represent those of their affiliated organizations, or those of the publisher, the editors and the reviewers. Any product that may be evaluated in this article, or claim that may be made by its manufacturer, is not guaranteed or endorsed by the publisher.

## References

[1] List T Wahlund K Larsson B. Psychosocial functioning and dental factors in adolescents with temporomandibular disorders: a case-control study. J Orofac Pain. (2001) 15:218–27. 11575192

[2] Ferreira CL Da Silva MA de Felicio CM. Orofacial myofunctional disorder in subjects with temporomandibular disorder. Cranio. (2009) 27:268–74. 10.1179/crn.2009.038 19891261

[3] Fisher E Heathcote LC Eccleston C Simons LE Palermo TM. Assessment of pain anxiety, pain catastrophizing, and fear of pain in children and adolescents with chronic pain: a systematic review and meta-analysis. J Pediatr Psychol. (2018) 43:314–25. 10.1093/jpepsy/jsx103 29049813 PMC6927870

[4] Kobayashi FY Gavião MBD Marquezin MCS Fonseca FLA Montes ABM Barbosa TS. Salivary stress biomarkers and anxiety symptoms in children with and without temporomandibular disorders. Brazilian Oral Res. (2017) 31:e78. 10.1590/1807-3107bor-2017.vol31.0078 29019550

[5] Feteih RM. Signs and symptoms of temporomandibular disorders and oral parafunctions in urban Saudi Arabian adolescents: a research report. Head Face Med. (2006) 2:25. 10.1186/1746-160X-2-25 16914032 PMC1563458

[6] Moyaho-Bernal A Lara-Munoz Mdel C Espinosa-De Santillana I Etchegoyen G. Prevalence of signs and symptoms of temporomandibular disorders in children in the State of Puebla, Mexico, evaluated with the research diagnostic criteria for temporomandibular disorders (RDC/TMD). Acta Odontol Latinoam. (2010) 23:228–33. 21638964

[7] Tecco S Crincoli V Di Bisceglie B Saccucci M Macrl M Polimeni A. Signs and symptoms of temporomandibular joint disorders in Caucasian children and adolescents. Cranio. (2011) 29:71–9. 10.1179/crn.2011.010 21370771

[8] Al-Khotani A Naimi-Akbar A Albadawi E Ernberg M Hedenberg-Magnusson B Christidis N. Prevalence of diagnosed temporomandibular disorders among Saudi Arabian children and adolescents. J Headache Pain. (2016) 17:41. 10.1186/s10194-016-0642-9 27102118 PMC4840132

[9] Poveda Roda R Bagan JV Diaz Fernandez JM Hernandez Bazan S Jimenez Soriano Y. Review of temporomandibular joint pathology. Part I: classification, epidemiology and risk factors. Med Oral Patol Oral Cir Bucal. (2007) 12:E292–8. 17664915

[10] Nilsson IM List T. Does adolescent self-reported TMD pain persist into early adulthood? A longitudinal study. Acta Odontol Scand. (2020) 78:377–83. 10.1080/00016357.2020.1730000 32073330

[11] Nilsson IM Drangsholt M List T. Impact of temporomandibular disorder pain in adolescents: differences by age and gender. J Orofac Pain. (2009) 23:115–22. 19492536

[12] Nilsson IM List T Willman A. Adolescents with temporomandibular disorder pain-the living with TMD pain phenomenon. J Orofac Pain. (2011) 25:107–16. 21528117

[13] Segu M Lobbia S Canale C Collesano V. [Quality of life in patients with temporomandibular disorders]. Minerva Stomatol. (2003) 52:279–87. 12874532

[14] Merlijn VPBM Hunfeld JAM van der Wouden JC Hazebroek-Kampschreur AAJM Passchier J Koes BW. Factors related to the quality of life in adolescents with chronic pain. Clin J Pain. (2006) 22:306–15. 10.1097/01.ajp.0000177509.93523.68 16514332

[15] Al-Khotani A Gjelset M Naimi-Akbar A Hedenberg-Magnusson B Ernberg M Christidis N. Using the child behavior checklist to determine associations between psychosocial aspects and TMD-related pain in children and adolescents. J Headache Pain. (2018) 19:88. 10.1186/s10194-018-0915-6 30242517 PMC6755608

[16] Simon DM Corbett BA. Examining associations between anxiety and cortisol in high functioning male children with autism. J Neurodev Disord. (2013) 5:32. 10.1186/1866-1955-5-32 24216056 PMC3827503

[17] Duggal S Carlson EA Sroufe LA Egeland B. Depressive symptomatology in childhood and adolescence. Dev Psychopathol. (2001) 13:143–64. 10.1017/S0954579401001109 11346049

[18] Barker MM Beresford B Bland M Fraser LK. Prevalence and incidence of anxiety and depression among children, adolescents, and young adults with life-limiting conditions: a systematic review and meta-analysis. JAMA Pediatr. (2019) 173:835–44. 10.1001/jamapediatrics.2019.1712 31282938 PMC6618774

[19] Da Silva Andrade A Gamero GH Pereira LJ Junqueira Zanin IC Gaviao MB. Salivary cortisol levels in young adults with temporomandibular disorders. Minerva Stomatol. (2008) 57:109–16. 10.3109/00016357.2010.494620 18427379

[20] Jastrowski Mano KE. School anxiety in children and adolescents with chronic pain. Pain Res Manag. (2017) 2017:8328174. 10.1155/2017/8328174 29081682 PMC5634599

[21] Sieber M Grubenmann E Ruggia GM Palla S. Relation between stress and symptoms of craniomandibular disorders in adolescents. Schweiz Monatsschr Zahnmed. (2003) 113:648–54. 10.5167/UZH-1504 12872589

[22] Pizolato RA Freitas-Fernandes FS Gaviao MB. Anxiety/depression and orofacial myofacial disorders as factors associated with TMD in children. Braz Oral Res. (2013) 27:156–62. 10.1590/S1806-83242013000100021 23538427

[23] Al-Khotani A Naimi-Akbar A Gjelset M Albadawi E Bello L Hedenberg-Magnusson B. The associations between psychosocial aspects and TMD-pain related aspects in children and adolescents. J Headache Pain. (2016) 17:30. 10.1186/s10194-016-0622-0 27044436 PMC4820412

[24] Al-Gelban KS. Depression, anxiety and stress among Saudi adolescent school boys. J R Soc Promot Health. (2007) 127:33–7. 10.1177/1466424007070492 17319315

[25] Nilsson IM List T Drangsholt M. The reliability and validity of self-reported temporomandibular disorder pain in adolescents. J Orofac Pain. (2006) 20:138–44. 16708831

[26] Lövgren A Parvaneh H Lobbezoo F Häggman-Henrikson B Wänman A Visscher CM. Diagnostic accuracy of three screening questions (3Q/TMD) in relation to the DC/TMD in a specialized orofacial pain clinic. Acta Odontol Scand. (2018) 76:380–6. 10.1080/00016357.2018.1439528 29448865

[27] Wahlund K List T Dworkin SF. Temporomandibular disorders in children and adolescents: reliability of a questionnaire, clinical examination, and diagnosis. J Orofac Pain. (1998) 12:42–51. 9656898

[28] Nilsson IM List T Drangsholt M. Prevalence of temporomandibular pain and subsequent dental treatment in Swedish adolescents. J Orofac Pain. (2005) 19:144–50. 15895837

[29] Goldfinger K Pomerantz AM. Psychological Assessment and Report Writing. SAGE Publications (2009). Available online at: https://us.sagepub.com/en-us/nam/psychological-assessment-and-report-writing/book237631

[30] Atia NS Kamel WW Fahmy HH. Effect of inter-parental conflict on adolescents' behavior in Zagazig City. Zagazig Nursing J. (2014) 10:149–39. 10.21608/ZNJ.2014.39188

[31] ASEBA. Translations. (2021). Available online at: aseba.org/translations/ (accessed April 21, 2021).

[32] Achenbach TM. Manual for the Child Behaviour Checklist: 4–18 Profile. Burlington VT, University of Vermont Dept of Psychiatry (1991).

[33] Ivarsson T Gillberg C Arvidsson T Broberg AG. The Youth Self-Report (YSR) and the Depression Self-Rating Scale (DSRS) as measures of depression and suicidality among adolescents. Eur Child Adolesc Psychiatry. (2002) 11:31–7. 10.1007/s007870200005 11942426

[34] Al-Harthy M Al-Bishri A Ekberg E Nilner M. Temporomandibular disorder pain in adult Saudi Arabians referred for specialised dental treatment. Swed Dent J. (2010) 34:149–58. 21121414

[35] Achenbach TM Rescorla LA. Manual for the ASEBA School-Age Forms and Profiles. Burlington, VT: University of Vermont, Research Center for Children, Youth, and Families (2001).

[36] Bonjardim LR Gaviao MB Pereira LJ Castelo PM. Anxiety and depression in adolescents and their relationship with signs and symptoms of temporomandibular disorders. Int J Prosthodont. (2005) 18:347–52. 16052791

[37] Karibe H Shimazu K Okamoto A Kawakami T Kato Y Warita-Naoi S. Prevalence and association of self-reported anxiety, pain, and oral parafunctional habits with temporomandibular disorders **i**n Japanese children and adolescents: a cross-sectional survey. BMC Oral Health. (2015) 15:8. 10.1186/1472-6831-15-8 25604542 PMC4324877

[38] Vanderas AP Voila P Papagiannoulis L. Urinary catecholamines as a measure of emotional stress in children with a digit-sucking habit: a preliminary study. ASDC J Dent Child. (2001) 68:179–82. 10.1007/978-1-4939-1726-6_59 11693009

[39] Ferrando M Andreu Y Galdon MJ Dura E Poveda R Bagan JV. Psychological variables and temporomandibular disorders: distress, coping, and personality. Oral Surg Oral Med Oral Pathol Oral Radiol Endod. (2004) 98:153–60. 10.1016/j.tripleo.2003.12.030 15316541

[40] Wahlund K List T Ohrbach R. The relationship between somatic and emotional stimuli: a comparison between adolescents with temporomandibular disorders (TMD) and a control group. Eur J Pain. (2005) 9:219–27. 10.1016/j.ejpain.2004.06.003 15737814

[41] Hinze V Crane C Ford T Buivydaite R Qiu L Gjelsvik B. The relationship between pain and suicidal vulnerability in adolescence: a systematic review. Lancet Child Adolescent Health. (2019) 3:899–916. 10.1016/S2352-4642(19)30267-6 31606322 PMC6842327

[42] Yap AU Tan KB Chua EK Tan HH. Depression and somatization in patients with temporomandibular disorders. J Prosthet Dent. (2002) 88:479–84. 10.1067/mpr.2002.129375 12473996

[43] Barlow JH Ellard DR. The psychosocial well-being of children with chronic disease, their parents and siblings: an overview of the research evidence base. Child Care Health Dev. (2006) 32:19–31. 10.1111/j.1365-2214.2006.00591.x 16398788

[44] Nadeau L Tessier R. Social adjustment of children with cerebral palsy in mainstream classes: peer perception. Dev Med Child Neurol. (2006) 48:331–6. 10.1017/S0012162206000739 16608539

[45] Roth-Isigkeit A Thyen U Stoven H Schwarzenberger J Schmucker P. Pain among children and adolescents: restrictions in daily living and triggering factors. Pediatrics. (2005) 115:e152–162. 10.1542/peds.2004-0682 15687423

[46] LeResche L Mancl LA Drangsholt MT Huang G Von Korff M. Predictors of onset of facial pain and temporomandibular disorders in early adolescence. Pain. (2007) 129:269–78. 10.1016/j.pain.2006.10.012 17134830 PMC1979093

[47] LeResche L Mancl LA Drangsholt MT Saunders K Von Korff M. Relationship of pain and symptoms to pubertal development in adolescents. Pain. (2005) 118:201–9. 10.1016/j.pain.2005.08.011 16213087

[48] Diatchenko L Slade GD Nackley AG Bhalang K Sigurdsson A Belfer I. Genetic basis for individual variations in pain perception and the development of a chronic pain condition. Hum Mol Genet. (2005) 14:135–43. 10.1093/hmg/ddi013 15537663

